# The Evaluation of North Wales SPiCE: Special Preparation in CASC Examination

**DOI:** 10.1192/bjo.2022.133

**Published:** 2022-06-20

**Authors:** Jiann Lin Loo, Catrin Thomas, Manjula Simiyon, Rahul Malhotra, Nikhil Gauri Shankar, Sarmishtha Bhattacharyya

**Affiliations:** 1 Betsi Cadwaladr University Health Board, Wrexham, United Kingdom.; 2 Betsi Cadwaladr University Health Board, Ruthin, United Kingdom; 3 Betsi Cadwaladr University Health Board, Llanfairfechan, United Kingdom

## Abstract

**Aims:**

As part of the effort to support core psychiatry trainees in North Wales to prepare for their CASC (Clinical Assessment and Skill Competency) exam, the North Wales SPiCE (Special Preparation in CASC Examination) Project has been initiated. This article aims to evaluate the SPiCE based on medical educational principles.

**Methods:**

A total of five candidates preparing for the CASC exam expressed interest and an organising committee was set up. Examiners consisted of a consultant and four specialist registrars while role players were recruited from non-exam sitting junior trainees. Five mock CASC stations were written and role-players were calibrated accordingly. The stations included: History taking for a patient with FTD (frontotemporal lobe dementia), MSE (Mental state examination) of a patient with mania and psychosis, explanation of CBT (cognitive-behavioural therapy), breaking bad news of NMS (neuroleptic malignant syndrome), and explanation of ECT (electroconvulsive therapy). The mock exam was conducted virtually using Microsoft Teams^TM^. The specialist registrars’ performances in feedback provision were assessed for their teaching using the AOT (Assessment of Teaching) form by the consultants. For core trainees who had played the part of organising committee members and role players, their volunteerism and educational management experience were assessed using the DONCS (Direct Observation of Non-clinical Skill) form by specialist trainees.

**Results:**

All five candidates passed all the stations (consists of both borderline pass, pass) in the mock exam with 25% improvements in confidence level were seen among candidates in four stations, i.e. ECT explanation, breaking bad news of NMS, CBT explanation, and MSE of a patient with mania and psychosis. All candidates feel the SPiCE programme was useful in helping their final preparation and they would recommend it to other candidates. Four of the candidates sat for the immediate CASC diet after the SPiCE received a pass result. All specialist registrars received positive AOT feedback for their teaching and all non-exam sitting junior trainees received positive DONCS feedback for their spirit of volunteerism and collaborative teamwork.

**Conclusion:**

The main strength of the SPiCE project is it utilises existing resources and volunteerism of the organising committee while its main limitation is it has only five stations rather than 16 stations in the real exam. Although the mock exam has improved the confidence of candidates and the majority of candidates pass the exam immediately after that, the causal link between the SPiCE and candidates’ results cannot be conclusively established given all candidates have a good baseline.

